# The Significance of Alliance Networks in Research and Development of Digital Health Products for Diabetes: Observational Study

**DOI:** 10.2196/32446

**Published:** 2021-10-21

**Authors:** Satoru Kikuchi, Kota Kodama, Shintaro Sengoku

**Affiliations:** 1 Department of Innovation Science School of Environment and Society Tokyo Institute of Technology Tokyo Japan; 2 Graduate School of Technology Management Ritsumeikan University Ibaraki Japan

**Keywords:** digital health, alliance, network, wearable device, diabetes

## Abstract

**Background:**

Digital health has been advancing owing to technological progress by means of smart devices and artificial intelligence, among other developments. In the field of diabetes especially, there are many active use cases of digital technology supporting the treatment of diabetes and improving lifestyle. In the innovation ecosystem, new alliance networks are formed not only by medical device companies and pharmaceutical companies, but also by information and communications technology companies and start-ups. While understanding and utilizing the network structure is important to increase the competitive advantage of companies, there is a lack of previous research describing the structure of alliance networks and the factors that lead to their formation in digital health.

**Objective:**

The aim of this study was to explore the significance of alliance networks, focusing on digital health for diabetes, in effectively implementing processes, from the research and development of products or services to their launch and market penetration.

**Methods:**

First, we listed the companies and contracts related to digital health for diabetes, visualized the change in the number of companies and the connections between companies in each industry, and analyzed the overview of the network. Second, we calculated the degree, betweenness centrality, and eigenvector centrality of each company in each year. Next, we analyzed the relationship between network centrality and market competitiveness by using annual sales as a parameter of company competitiveness. We also compared the network centrality of each company by industry or headquarters location (or both) and analyzed the characteristics of companies with higher centrality. Finally, we analyzed the relationship between network centrality and the number of products certified or approved by the US Food and Drug Administration.

**Results:**

We found the degree centrality of companies was correlated with an increase in their sales. The betweenness and eigenvector centralities of medical devices companies located in the United States were significantly higher than those outside the United States (*P*=.04 and .005, respectively). Finally, the degree, betweenness, and eigenvector centralities were correlated with an increase in the number of Class III, but not of Class I nor II, medical device products.

**Conclusions:**

These findings give rise to new insights into industry ecosystem for digital health and its requirement and expect a contribution to research and development practices in the field of digital health.

## Introduction

### Background

According to the US Food and Drug Administration (FDA), the scope of digital health covers categories such as mobile health (mHealth), health information technology, wearable devices, telehealth and telemedicine, and personalized medicine [1]. Digital health involves the use of sensors, software, connectivity, and computing platforms. These technologies have been used across a range of medical applications and wellness applications; in medical applications, they can be used as medical products themselves or separately added to medical products. They can also be used for research and development of medical products [1]. Digital health aims to reduce inefficiencies in health care services and costs, improve access to health care services and their quality, and promote personalized medicine. Digital tools are expected to aid disease prevention, early diagnosis, and appropriate management of chronic diseases, along with providing opportunities to improve health care outcomes and increase efficiency by enabling patients to access their own data, gain a holistic view of their health status, and take control of their own health.

Another expected utility of digital health is lean innovation or increased cost efficiency. Currently, health care costs are increasing in developed countries, accounting for more than 10% of gross domestic product; reducing health care costs has been a major issue. In addition, the number of patients with chronic diseases such as diabetes is expected to increase due to accelerated aging of the population, which would further increase health care costs [2]. Furthermore, in emerging countries, there is a lack of medical and health care services, and digital health is expected to be a solution to this problem.

The use of digital health is increasing with regard to diabetes mellitus. Diabetes is a chronic disease characterized by elevated levels of blood glucose, which causes serious damage to the heart, blood vessels, eyes, kidneys, and nerves. The treatment and management of diabetes differs from those of other diseases in that it requires medical devices such as syringes, insulin pens, insulin pumps, for drug administration; blood glucose monitoring devices such as continuous glucose monitoring and flash glucose monitoring for disease management; and lifestyle guidance such as diet and exercise, which can be managed by the use of mobile apps [3]. In the digital health for diabetes space, in addition to the existing health care companies, such as medical device companies and pharmaceutical companies, new players, such as app service providers and data management solution providers, are being engaged, according to research2guidance [4]. However, the relationships among companies, that is, what roles each plays and how each partner contributes to digital health, are not very well understood.

Understanding alliance networks among companies is critical for predicting future developments in digital health. The goal is to integrate digital technology with, and not make it a substitute for, health care providers. To realize this, existing electronic medical records and treatment and management methods need to be integrated with digital data, and systems need to be built so that hardware can be interoperable. Digital therapy platforms are expected to play a central role in supporting diabetes treatment and self-management through embedded algorithms [5]. As part of the new system, existing health care companies, including medical device companies, will need to partner with companies that own digital therapy platforms, or simply technology companies, and form a network centered on technology companies.

In our previous research, we focused on the research about technology companies with high network centralities in the alliance network about digital health for diabetes, and characterized them into 3 business models: (1) intermediary model, (2) substitute model, and (3) direct-to-consumer model [6]. As the next step, in this research, we provide an overview of the structure of the alliance network and its time change, and factors that lead to their formation in digital health for diabetes.

### Research Objectives and Hypothesis

The significance and utility of alliance networks have been discussed in many previous studies. Companies can form alliances according to their strategic intentions and actions, and benefit from access to and exchange of information through their networks [7]. It has been pointed out that alliance networks can promote information diffusion, innovation, and learning in companies [8,9], and that they can change the flow of information and knowledge and affect the competitive advantage of companies [10]. Therefore, companies located at the center of an alliance network can disseminate information and knowledge, and act as a gateway for information exchange, making a bigger impact as compared with other firms in the network. Some studies have also linked firm performance to network centrality, noting that the formation of new networks facilitates collective knowledge sharing and exploratory learning in new technological domains [9,11].

Previous research studies have shown that facilitating learning in alliance networks is important in new technology domains, and that being centrally located in such networks can lead to increased competitive advantage for companies [7-12]. In drug development including antibody, cell therapy, gene therapy, and personalized medicine, increase of external collaborations has been observed [13]. Wherever new technologies are used in digital health, it is assumed that alliance networks are built, and that learning and performance are improved through them. In this study, we examined the relationship between network centrality and competitiveness by using the increase in total annual sales as a parameter to show an increase in competitiveness.

Hypothesis 1: The more central a company is in an alliance network, the more competitive it is in the market.

Regarding the use of digital health for diabetes, Kerr et al [5] predicted a future ecosystem in which digital therapeutic platforms are at the center. While technology companies are capable of platform-based horizontal specialization, pharmaceutical and medical device companies need vertical integration because their products are approved individually [14]. Considering the above, it is assumed that a network centered on technology companies is being formed in the digital health of diabetes.

In addition to the attributes of the companies being important in digital health, their geographic location may also be consequential, because many US companies have a significant presence. The number of guidelines on digital health issued by the regulatory authorities in each region from 2005 to 2020 was 21 from the FDA, 1 from the European Medicines Agency (EMA), and 0 from the Japan Pharmaceuticals and Medical Devices Agency (PMDA) as of October 2020 [15,16]. This suggests that the United States may be the country most likely to develop and launch products related to digital health. In today’s globalized world, any company from any country can develop a product in the United States. However, because it could be thought that geographical proximity to the FDA would be advantageous in negotiations with the FDA, and it was assumed that companies headquartered in the United States would likely be better positioned in the alliance network, we hypothesized the following.

Hypothesis 2: Companies with high network centrality are characterized as technology companies and companies headquartered in the United States.

Finally, we examined the relationship between network centrality and the profile of the digital health products and services that these companies are engaged in. Digital health is characterized by its ability to handle big data. Medical devices can be broadly classified into those that are used only when necessary and those that are worn at all times, such as wearable devices, the former being classified as Class II medical devices, and the latter as Class III. In the digital health sector, Class III medical devices are thought to be used because they can obtain a large amount of data 24/7 by connecting to wearable devices.

While it has been reported that about 21% of users abandon mobile apps after one use, with retention of users being a challenge [5], when an app is connected to a wearable device, data transfer and other activities are performed passively, even if the user does not actively use the app. Therefore, for continuous data collection, products accompanied by wearable devices are likely to become more mainstream as compared with mobile apps alone. Here, a product with a mobile app falls under Class II, while a product connected to a wearable device falls under Class III. Considering the above, it is assumed that Class III products are likely to be the main battleground for leading companies.

Hypothesis 3: Companies with high network centrality are more likely to have Class III products as compared to those with Class I and II products.

## Methods

We used the data set which we made in the previous research [6]. We listed 57 companies that were engaged in diabetes digital health based on public information [4,17,18]. Next, we listed their alliance partnerships in diabetes digital health from their press releases. The partnerships we listed covers not only simple contracts such as collaboration agreement, financial agreement, commercial agreement, and patent license agreement, but also joint venture, merger and acquisition, and Precertification (Pre-Cert) Pilot Program by the FDA as one of the styles of partnership. New companies that appeared as partners were added to the list, and the listings of partnerships of these companies were repeated in the same way until no new companies appeared. As a result, 231 companies and 331 contracts were listed [6]. We listed information from Crunchbase [19] for company name, year of establishment, country of headquarters, company website link, and Bloomberg [20] for sector and industry affiliation. The sector and industry information were taken directly from Bloomberg [20]. The listing included contracts that were released until August 13, 2020 [6].

For the number of FDA approvals, we used the Premarket Approval (PMA) database for Class III [21] and the 510(k) database for Classes I and II [22]. The number of FDA approvals of medical devices related to diabetes from 2005 to 2020 by the company was listed.

For network analysis, we used the open software package Gephi 0.9.2 [23], with the companies collected as nodes and the contracts collected used as edges. Thereafter, for each company, we calculated and extracted the degree, betweenness centrality, and eigenvector centrality as network parameters from 2011 to 2020 using Gephi 0.9.2. The definitions of the 3 network parameters were as follows: the degree, the number of edges connected to the node; the betweenness centrality, the number of times a node lies on the shortest path between other nodes; and the eigenvector centrality, the node’s influence based on the number of links it has to other nodes in a network.

## Results

### Number of Players and Contracts in Digital Health for Diabetes

**Figure 1:**
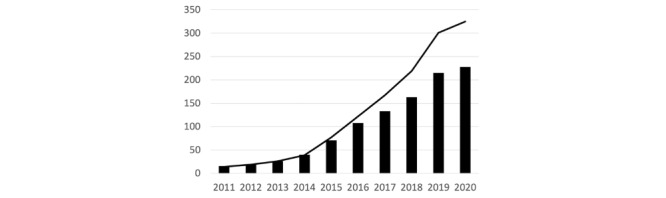
Number of companies and contracts in the diabetes alliance network (year 2011-2020). Each bar represents the number of companies within the scope, while the line represents the number of contracts over time.

The number of companies and the number of contracts related to diabetes digital health at each point in time from 2011 to 2020 (until August 13, 2020) are shown in Figure 1. The number of companies and contracts are found to have increased slightly from 2011 to 2014, rapidly after 2015, and reached 228 and 325 in 2020, respectively.

### Network Structure and Components Over Time

**Figure 2:**
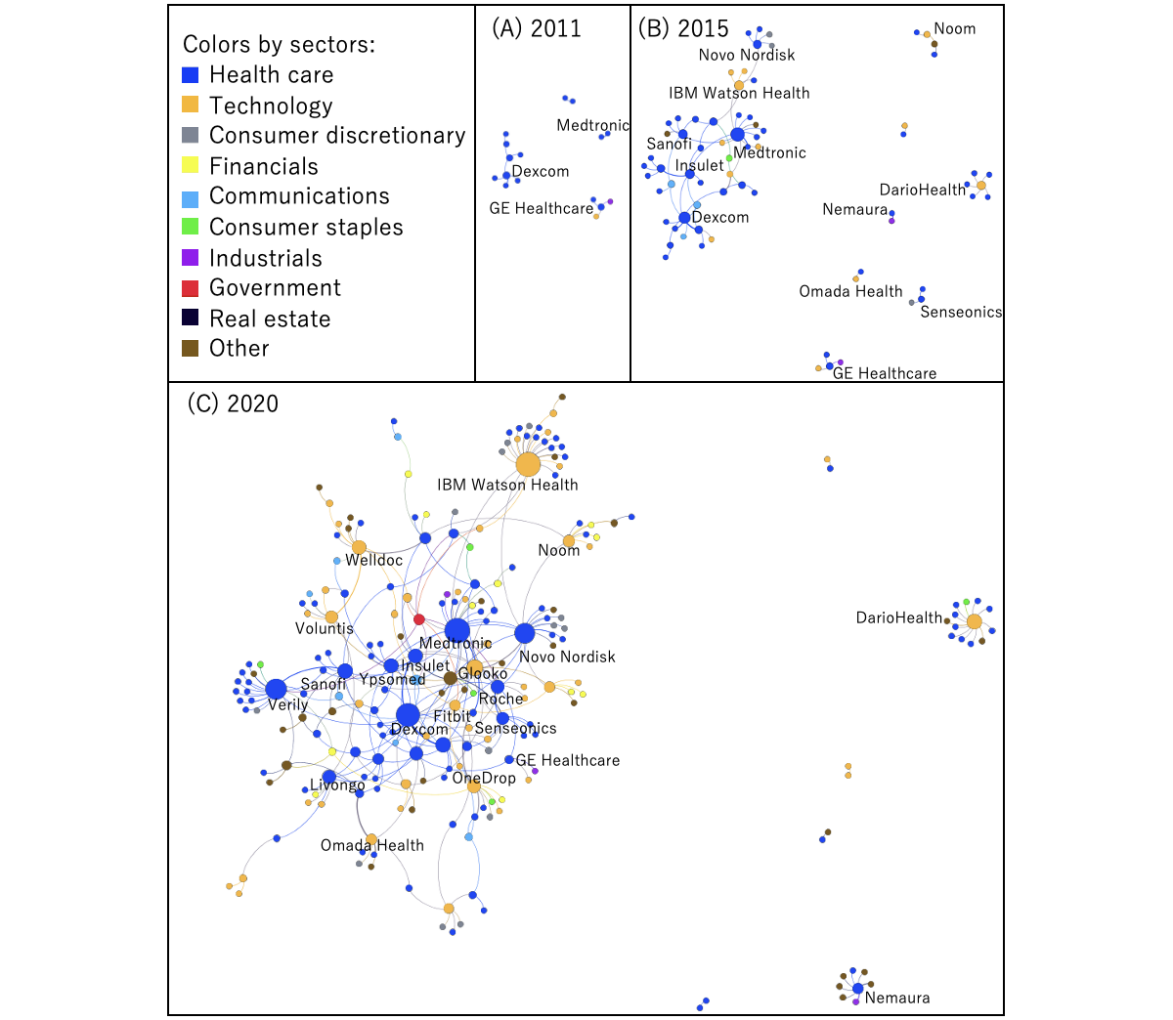
Changes in the alliance network for digital health in diabetes. Network in (A) 2011, (B) 2015, (C) 2020. Each label represents the centered company name(s) in a cluster.

To observe the changes in the connections between players in the alliance network for diabetes digital health, we drew the networks in 2011, 2015, and 2020, using node as the player, edge as the contract, and color coding by the sector to which the player belongs (Figure 2). The alliance network in 2011 was drawn as a representative of the embryonic phase of digital health, in 2015 as the start of growth phase, and in 2020 as the latest.

In 2011, contracts were mainly made by health care companies (medical equipment and devices, and biotech and pharma). In 2015, the number of technology companies increased and they started to connect to health care companies, including health care facilities; some communications companies (eg, Google, Tidepool) entered the network and connected to health care companies. In 2020, the number of technology companies increased further; companies from various sectors, including consumer discretionary services (mainly universities), entered the network, and health care and technology companies worked as hubs in this network.

### Relationship Between Network Centrality and Companies’ Total Annual Sales

**Figure 3:**
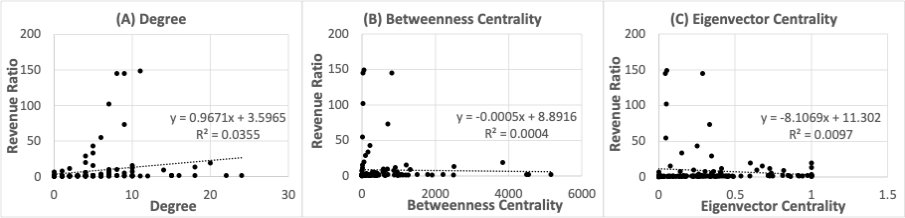
Scatter plot of network centrality and annual growth in sales.

To investigate the relationship between network centrality and the total annual sales of the companies (hypothesis 1), we selected 16 companies with a degree higher than 4 in 2020, and annual reports published from 2011 to 2020. Of the 16 companies, 7 were medical devices companies, 7 were pharmaceutical companies, and 2 were technology companies. We then examined the relationship between the degree and annual gross sales ratio, and only the degree was correlated with the sales ratio (r=0.188, *P*<.03; Figure 3).

**Figure 4:**
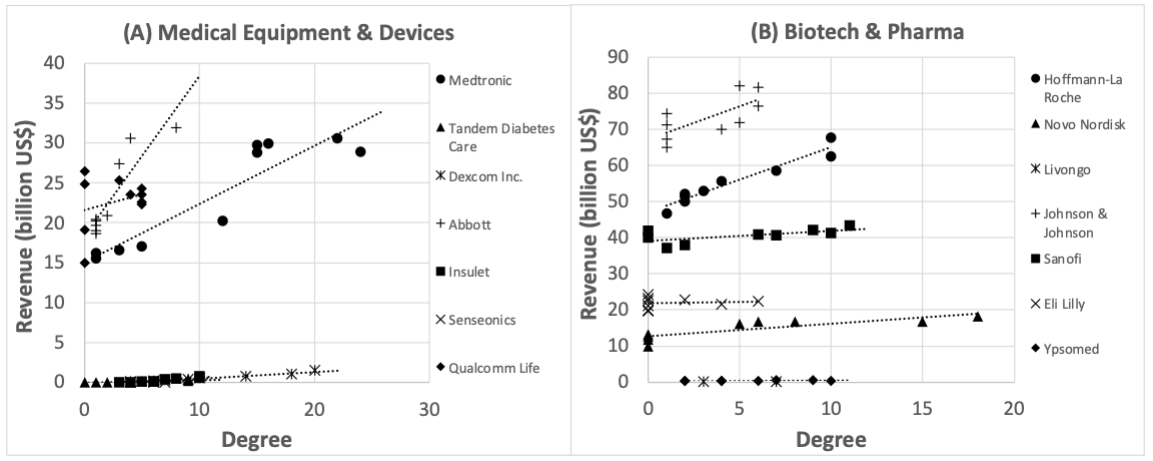
Scatter plot of degree and annual sales.

We then examined the relationship between the degree and sales, focusing only on medical devices and pharmaceutical companies. As for the medical equipment and devices, and biotech and pharma companies, 5 out of 7 showed a positive and significant correlation (Figure 4, Table 1).

Based on these results, an increase in sales for centered companies was confirmed for degree centrality. At the individual company level, 5 of the 7 medical equipment and devices, and biotech and pharma companies showed a positive and significant correlation. Therefore, hypothesis 1 was confirmed for degree centrality and selected company cases in medical equipment and devices and biotech and pharma sectors.

**Table 1:** *t* test of relationship between degree and annual sales in each company.

Sector and companies			n		r	*t* value (*df*)		*P* value
**Medical equipment and device**								
	Medtronic	10		0.920		6.66 (8)	<.001	
	Tandem Diabetes Care	9		0.888		5.10 (7)	.001	
	Dexcom Inc.	9		0.981		13.4 (7)	<.001	
	Abbott	9		0.904		5.59 (7)	<.001	
	Insulet	9		0.927		6.56 (7)	<.001	
	Senseonics	3		0.932		2.56 (1)	.24	
	Qualcomm Life	10		0.283		0.835 (8)	.43	
**Biotech and Pharma**								
	Hoffmann-La Roche	9		0.965		9.72 (7)	<.001	
	Novo Nordisk	9		0.842		4.12 (7)	.004	
	Johnson & Johnson	9		0.706		2.64 (7)	.03	
	Ypsomed	8		0.857		4.07 (6)	.006	
	Sanofi	9		0.631		2.15 (7)	.07	
	Eli Lilly	9		0.0856		0.227 (7)	.83	
	Livongo	2		NA^a^		NA^a^	NA^a^	

^a^NA: not applicable.

### Characteristics of Companies With High Network Centricity

**Figure 5:**
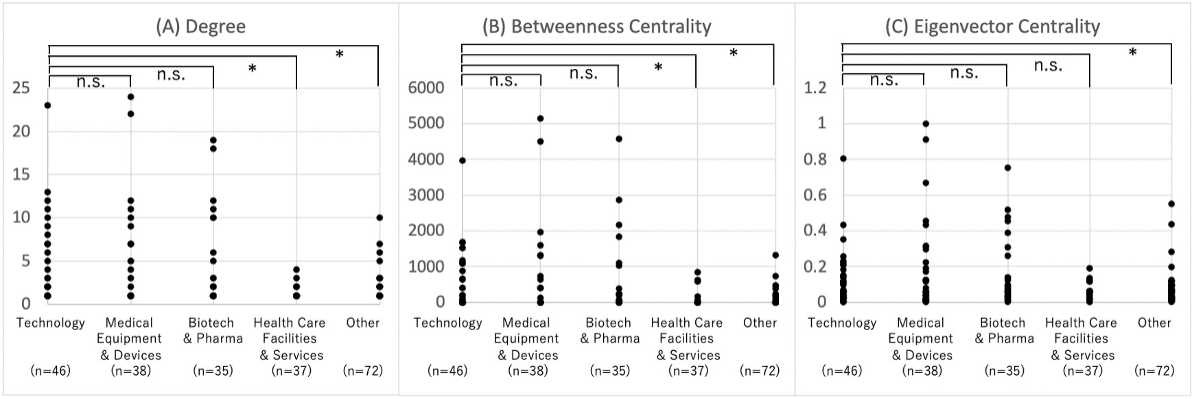
Comparison of network parameters by industry. *P<.05, n.s.: not significant.

Companies in the network in 2020 were classified into medical device companies, health care facilities, pharmaceutical companies, and technology companies, and their network parameters were compared (Figure 5). It was confirmed that technology companies were significantly higher than health care facilities in terms of degree, betweenness centrality, and others in all indicators (*P*<.001 and .001 for degree and betweenness centrality, respectively). It was also confirmed that technology companies were not significantly different from medical device companies or pharmaceutical companies in these indicators (*P*<.001 for degree and betweenness, and *P*=.03 for eigenvector centrality, respectively). These results suggest that technology companies are located at the center of the alliance network as well as medical device companies and pharmaceutical companies.

**Figure 6:**
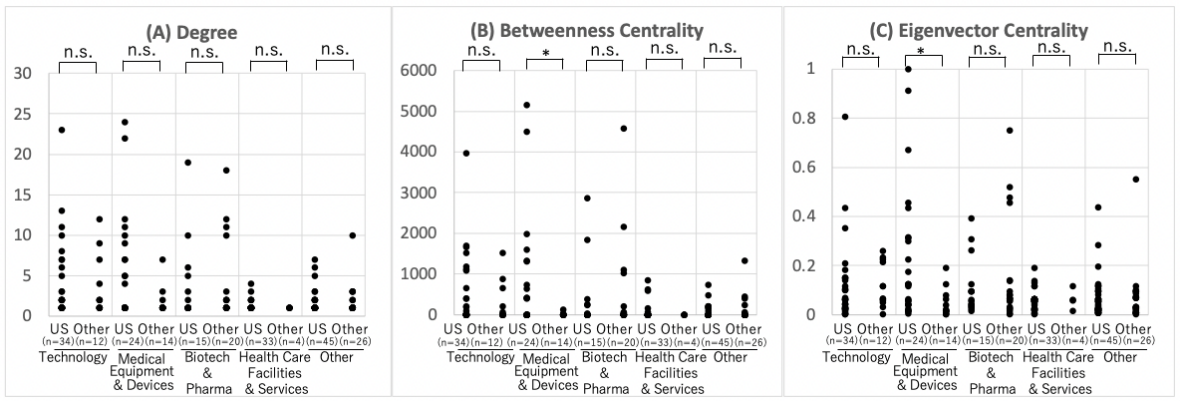
Comparison of network parameters by industry and region. *P<.05, n.s.: not significant.

Next, we focused on regional and industry classifications and categorized companies according to the location of their operational headquarters in 2020 into the United States and other countries (Figure 6). No significant differences in degree, betweenness centrality, and eigenvector centrality were found between companies in the United States and other countries, except for the betweenness (*P*=.035) and eigenvector centrality (*P*=.005) of medical equipment and device companies.

From these results for hypothesis 2, we confirmed that technology companies have high network centrality, as do medical devices and pharmaceutical companies, and, in particular, medical device companies based in the United States.

### Relationship Between Network Centrality and the Number of FDA-Approved Products of a Company

To confirm the relationship between network centrality and the number of products of a company (hypothesis 3), we examined the number of FDA approvals for diabetes-related medical devices (Class I, II, and III) from 2005 to 2020 and the number of FDA approvals by company. In PMA, 17 products (6 companies) were approved for diabetes. We could use all 6 companies for our analysis because all had at least one degree in the network. In 510(k), 568 products (148 companies) were cleared for diabetes. For simplicity, we selected 32 companies whose cumulative number of approvals was more than 3 in the timeframe from 2005 to 2020. Next, we selected companies with a degree of at least one in the network, after which 7 out of 32 companies remained.

**Figure 7:**
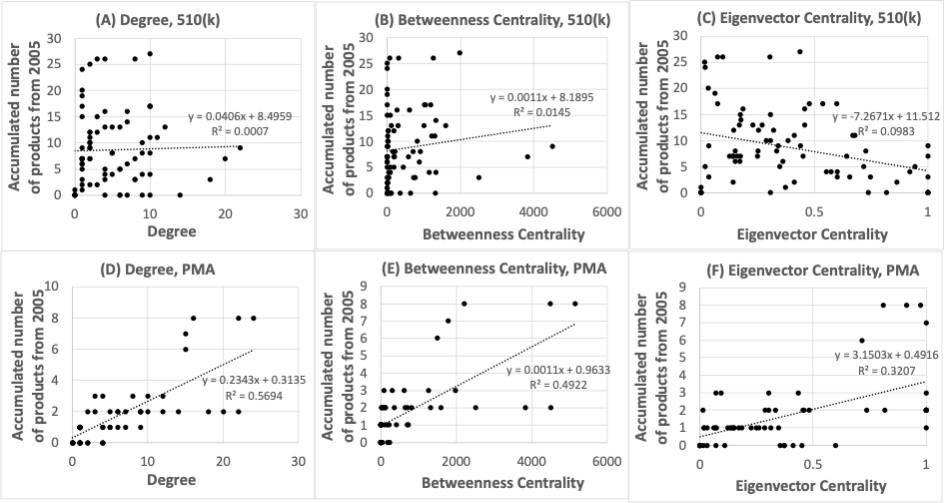
Scatter plot of network centrality and the number of 501(k) products or PMA products. PMA: premarket approval.

There was a relationship between network centralities (ie, degree, betweenness centrality, and eigenvector centrality) and the cumulative number of FDA approvals, as PMA showed a significant correlation (*P*=3.33×10^–12^, 4.27×10^–10^, and 2.40×10^–6^, respectively), whereas there was no correlation found for 510(k) (Figure 7). These results suggest that companies with high network centrality are more likely to have Class III products as compared to those with Class I and II products.

## Discussion

### Principal Findings

This paper is the first to highlight the importance of studying the business strategies of distinctive companies by focusing on network centrality and aims to contribute to the creation of innovative digital health products and services. The structure of the alliance network and its time change, and factors that lead to their formation in digital health for diabetes were observed.

It was confirmed that the higher the degree of a company’s alliance network, the greater the increase in sales of the company. One reason for this is the possibility that the degree increases with an increase in sales; in general, as the size of a company increases, its presence increases, and it becomes easier to invest in the next business, thus expanding the opportunities for alliances with other firms. The other is the possibility that an increase in the degree of new alliances leads to the development and sale of new products and services, which in turn increase total annual sales.

In the alliance network, technology companies were located at the center of the alliance network as well as medical device companies and pharmaceutical companies. In terms of regions, medical device companies in the United States showed higher betweenness and eigenvector centrality than the companies in other countries. This indicates that medical device companies in the United States are more connected to firms with high network centrality as compared with those in other countries. As mentioned in the “Introduction” section, the reason for this may be the geographical proximity to the FDA, which promotes digital health through the issuance of guidelines.

Furthermore, companies with higher network centralities have a higher number of Class III FDA-approved products. This can be attributed to 2 possible causal relationships: (1) new alliances may have enabled a firm to gain the ability to develop Class III products, or (2) the possession of new Class III products may have led to new alliances, or both. Product-based case studies and time-series analyses are necessary to elucidate this mechanism.

**Figure 8:**
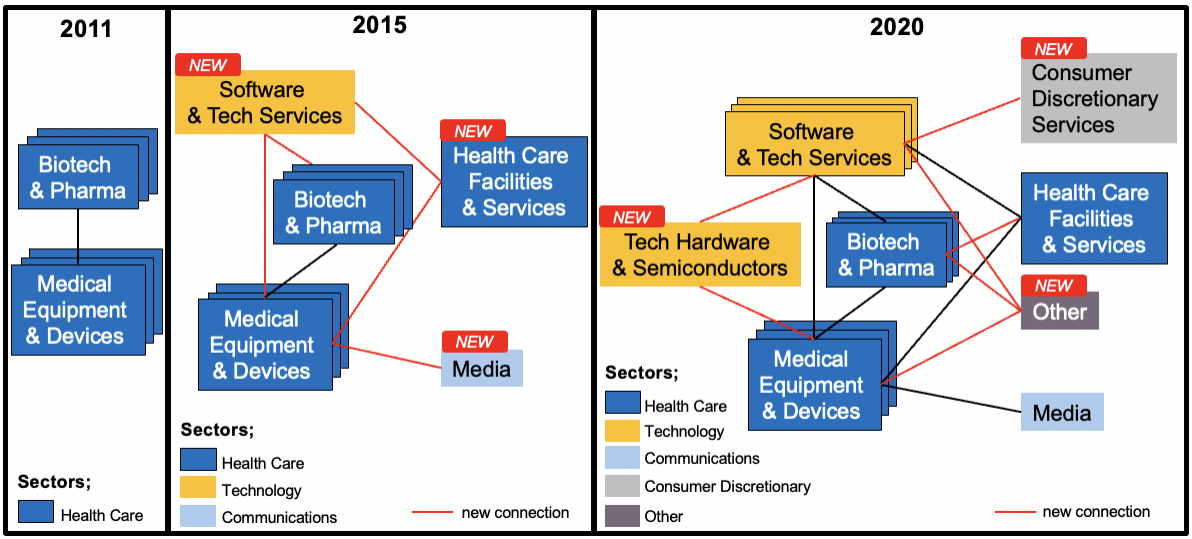
Overview of industry ecosystems and historical change.

A schematic diagram of the ecosystem transition from 2011 to 2020 is shown in Figure 8. In the observed evolution of the alliance networks for digital health in diabetes, the key actors used to be incumbent companies in 2011, and a diverse range of companies participated, creating an ecosystem different from that of the traditional health care industry. In particular, the presence of technology companies is growing and has the potential to drive paradigmatic innovation in digital health.

### Limitations

First, this study focused on diabetes. Because digital technologies are used in the treatment and management of diabetes, we used the case study of digital health for diabetes. The findings of this study might be limited to the field of diabetes, and a more detailed study is needed to elucidate the innovation mechanism behind it. For example, there might be a different feature in the digital health for psychiatry (eg, a digital biomarker to assess the efficacy in patients).

Second, the contracts and companies which are listed for this study are limited to information that was publicly available in the press releases of the companies by August 13, 2020. Because the contracts about digital health are increasing drastically in recent years and digital health was accelerated due to the COVID-19 pandemic in 2020, the findings in this study might be just a snapshot until August 13, 2020.

And finally, a cluster analysis in the alliance network was not implemented. In this study, the overall picture of the Diabetes Alliance Network was analyzed. By contrast, as 14 clusters have been identified in the network as of 2020, new insights may be gained by conducting cluster analysis. For example, it may be possible to classify clusters as some aiming at personalized diabetes care and some aiming at diabetes prevention, and each cluster may have its own unique characteristics.

### Comparison With Prior Work

To our knowledge, this is the first study to highlight the importance of studying the business strategies of distinctive companies by focusing on network centrality and aims to contribute to the creation of innovative digital health products and services.

This paper is aligned with the past literature which showed that facilitating learning in alliance networks is important in new technology domains, and that being centrally located in such networks can lead to increased competitive advantage for companies [7-12]. In this study, the alliance network is growing in digital health for diabetes, and it was confirmed that the higher the degree of a company’s alliance network, the greater the increase in sales of the company.

In our previous research, we listed the technology companies with high network centralities in the alliance network about digital health for diabetes, and characterized them into 3 business models: (1) intermediary model, (2) substitute model, and (3) direct-to-consumer model [6]. The study focused on the technology companies. By contrast, this study, for the first time, presents an overview of the structure of the alliance network and its time change, and factors that lead to its formation in digital health for diabetes.

### Conclusions

In this study, we focused on digital health for diabetes and analyzed the structural search of alliance networks and the factors affecting their structure formation. We found that the degree in the alliance network was correlated with the growth rate of sales, whereas the betweenness and eigenvector centralities were not, suggesting that the network centrality may not affect the companies’ sales. In addition, medical device companies in the United States had a higher betweenness and eigenvector centrality than those of others, implying the contribution of closer proximity to the FDA that had been proactively establishing related guidelines and encouraging new entrants to digital health. Furthermore, network centralities were correlated with an increase in the number of Class III products but not of Class I nor II products, suggesting that currently, the higher network centrality may matter to products with potentially higher risks.

This is the first study to highlight the importance of studying the business strategies of distinctive companies by focusing on network centrality and aims to contribute to the creation of innovative digital health products and services.

## Abbreviations

EMA: European Medicines Agency
FDA: Food and Drug Administration
mHealth: mobile Health
PMA: premarket approval
PMDA: Pharmaceuticals and Medical Devices Agency
